# Treatment of In-Stent Restenosis by Excimer Laser Coronary Atherectomy and Drug-Coated Balloon: Serial Assessment with Optical Coherence Tomography

**DOI:** 10.1155/2019/6515129

**Published:** 2019-04-18

**Authors:** Toru Miyazaki, Takashi Ashikaga, Taku Fukushima, Yu Hatano, Taro Sasaoka, Ken Kurihara, Yuichi Ono, Shigeo Shimizu, Kenichiro Otomo, Kenzo Hirao

**Affiliations:** ^1^Department of Cardiovascular Medicine, Ome Municipal General Hospital, Tokyo, Japan; ^2^Department of Cardiology, Musashino Red Cross Hospital, Tokyo, Japan; ^3^Department of Cardiovascular Medicine, National Disaster Medical Center, Tokyo, Japan; ^4^Department of Cardiovascular Medicine, Tokyo Medical and Dental University, Tokyo, Japan

## Abstract

**Objectives:**

We aimed to compare the results of neointimal modification before drug-coated balloon (DCB) treatment with excimer laser coronary atherectomy (ELCA) plus scoring balloon predilation versus scoring balloon alone in patients presenting with in-stent restenosis (ISR).

**Background:**

Treatment of ISR with ELCA typically results in superior acute gain by neointima debulking. However, the efficacy of combination therapy of ELCA and DCB remains unknown.

**Methods:**

A total of 42 patients (44 ISR lesions) undergoing DCB treatment with ELCA plus scoring balloon (ELCA group, n = 18) or scoring balloon alone (non-ELCA group, n = 24) were evaluated via serial assessment by optical coherence tomography (OCT) performed before, after intervention, and at 6 months.

**Results:**

Although there was significantly greater frequency of diffuse restenosis and percent diameter stenosis (%DS) after intervention in the ELCA group, comparable result was shown in %DS, late lumen loss, and binary angiographic restenosis at follow-up. On OCT analysis, a decreased tendency in the minimum lumen area and a significant decrease in the minimum stent area were observed in the ELCA group between 6-month follow-up and after intervention (-0.89 ± 1.36 mm^2^ vs. -0.09 ± 1.25 mm^2^, p = 0.05, -0.49 ± 1.48 mm^2^ vs. 0.28 ± 0.78 mm^2^, p = 0.03, respectively). The changes in the neointimal area were similar between the groups, and target lesion revascularization showed comparable rates at 1 year (11.1% vs. 11.4%, p = 0.85).

**Conclusions:**

Despite greater %DS after intervention, ELCA before DCB had possible benefit for late angiographic and clinical outcome.

## 1. Introduction

In-stent restenosis (ISR) resulting from neointimal hyperplasia remains a major limitation after stent implantation. Repeat stenting with drug-eluting stents (DES) is considered as the mainstay for the treatment of ISR [1]. Recently, drug-coated balloon (DCB) angioplasty for ISR was considered as an alternative treatment strategy instead of DES implantation because of its ability to provide an opportunity in cases of reinterventions and as it is associated with favorable results without adding a new stent [2–6]. In clinical practice, the modification of neointima provides superior acute gain and may be achieved by cutting or scoring balloons as well as rotational atherectomy during percutaneous coronary intervention (PCI). In particular, excimer laser coronary atherectomy (ELCA) is thought to be advantageous for the treatment of ISR by neointima debulking. Compared with plain old balloon angioplasty (POBA), ELCA plus POBA improved lumen dimensions by a combination of tissue ablation, tissue extrusion, and additional stent expansion [7, 8]. However, ELCA with DCB treatment has not demonstrated clear clinical benefits. Therefore, the aim of this study was to compare the results of neointimal modification before DCB treatment with ELCA plus scoring balloon predilation versus scoring balloon alone in patients presenting with ISR.

## 2. Methods

This study was a physician-initiated, two-center retrospective study designed to describe the characteristics and assess serial changes induced by the DCB in ISR lesions. Between March 2014 and May 2018, a total of 42 patients treated with DCB (SeQuent® Please, Nipro, Japan), with 44 ISR lesions (including 30 DES) that were evaluated by frequency-domain optical coherence tomography (FD-OCT) (ILUMIEN OPTIS™; Abbott Vascular, Inc., 3200 Lakeside Drive, Santa Clara, California, USA) were included in this study. All procedures were performed according to standard clinical guidelines. Patients with cardiogenic shock, acute ST-segment elevation myocardial infarction (MI), target lesions located in the left main stem, malignancies or other comorbid conditions associated with a short life expectancy, contraindications to antiplatelet therapy and paclitaxel, or pregnancy were considered ineligible for the study. Acute coronary syndrome (ACS) was defined here as unstable angina pectoris or non–ST-segment elevation MI. All patients received standard medications, including 81-100 mg aspirin, and 75 mg clopidogrel, 3.75 mg prasugrel, or 200 mg ticlopidine. Patients presenting with ACS received aspirin 200 mg and a clopidogrel loading dose of 300 mg or prasugrel of 20 mg before the procedure. Bivalirudin or glycoprotein IIb/IIIa antagonist was not used in this study. A regimen of dual antiplatelet therapy was maintained for at least 6 months if it was well tolerated by patients without risk for bleeding. Cardiac medications were prescribed according to the judgment of each patient's physician. The PCI strategy of ISR before DCB treatment was dependent on each operator. During the procedure, patients were administered intravenous heparin with a target activated clotting time of 250–300 s. A total of 18 patients underwent DCB angioplasty with ELCA plus scoring balloon predilation (ELCA group, n = 18). Twenty-four patients underwent DCB angioplasty with scoring balloon predilation (non-ELCA group, n = 24). Lacrosse® nonslip element (NSE) ALPHA (Goodman Co., LTD., Nagoya, Japan) was used as a scoring balloon. If residual stenosis was more than 50% after the lesion modification procedure, the operator increased the dilating pressure or used a noncompliant balloon. A DCB with a size similar to that of the previous scoring balloon was selected. The length of the DCB was chosen such that it overlapped with the lesion compared with a scoring balloon. Angiographic success was defined as achievement of final residual stenosis <30% (by visual estimate) and Thrombolysis. In myocardial infarction flow grade 3, procedural success was defined as angiographic success without the occurrence of in-hospital major adverse cardiac events. Acute procedural results were evaluated by quantitative coronary angiography (QCA) and FD-OCT. Follow-up angiography, QCA, and OCT analyses were performed in all patients at 6 months after initial ISR treatment. If a target lesion revascularization (TLR) occurred before 6-month follow-up, the patient was excluded from follow-up angiographic and OCT analysis. This study was approved by the institutional ethical review board at the Tokyo Medical and Dental University and performed according to Ethical Guidelines for Epidemiological Research. We published all relevant details of this study instead of obtaining informed consent.

### 2.1. Excimer Laser System

A pulsed xenon chloride excimer laser with a wavelength of 308 nm was utilized as the laser source (CVX-300; Spectranetics, Colorado Springs, CO, USA). Pulse duration was 135 ns and output was 200 mJ/pulse. Laser energy was delivered through concentric multifiber catheters (Vitesse C, Spectranetics) with a diameter of 1.4 mm. Energy parameters were initially set at a fluency of 45 mJ/mm^2^ and a repetition rate of 25 Hz. The guiding catheter was filled with saline immediately before lasing. The operator then initiated lasing, advancing the laser catheter at a speed of 0.5 mm/s while an assistant flushed saline 2–3 mL/s [9]. The operator could raise the fluency and repetition rate to 60 mJ/mm^2^ and 40 Hz maximum if the neointimal hyperplasia was not adequately debulked.

### 2.2. Angiographic Analysis

QCA analysis was performed using the CASSII software (Pie Medical Imaging, Maastricht, Netherlands) or QCA-CMS software (Medis Medical Imaging Systems, Leiden, and the Netherlands). The minimum lumen diameter (MLD), reference diameter (RD), defined as average diameter of proximal and distal healthy segments, percent diameter stenosis (%DS), and lesion length that were measured in diastolic frames from orthogonal projections were compared between the ELCA and the non-ELCA groups. All patients were scheduled to undergo clinical and angiographic follow-up at 6 months. Late lumen loss (LLL) was defined as the difference between the MLD immediately after the procedure and at the 6-month follow-up angiography. Late lumen enlargement (LLE) was defined as a having late lumen gain. Binary restenosis was defined as %DS ≥50% at follow-up angiogram.

### 2.3. OCT Analysis

**Figure 1:**
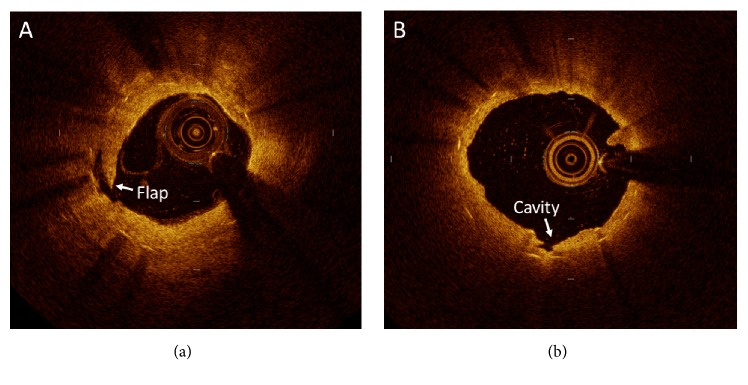
*Morphology of dissections:* (a) flap dissections and (b) cavities.

FD-OCT imaging catheter was inserted and advanced distal to the lesion of each ISR. Blood clearance was measured by the injection of contrast dye or low molecular weight dextran directly through the guiding catheter. Cross-sectional OCT images were analyzed at 1-mm intervals for ISR segment with a length of at least 5 mm at the proximal and distal margins dilated with a DCB. Measurement of minimum lumen cross-sectional area (MLCSA) and minimum stent cross-sectional area (MSCSA), including metallic strut, was performed. The neointimal cross-sectional area (CSA) was calculated as the stent CSA minus the luminal CSA, and percentage of neointimal CSA was calculated as the neointimal CSA/stent CSA×100. These cross sections were serially matched with those after PCI and at follow-up using landmarks, such as stent edges or bifurcations. Differences in the change in the MLSCSA, MSCSA, and neointimal CSA between after and before PCI, and between at follow-up and after PCI were evaluated. Dissection was defined as disruption of the vessel luminal surface, including flaps and cavities [10], in at least two consecutive cross-sectional images (Figure 1). The number of dissections after PCI was added, and the change in the number of neointimal dissections was analyzed at 6 months of follow-up.

### 2.4. Study Endpoints

The primary endpoint of this study was in-segment LLL. The secondary endpoint was a reduction in the neointimal area assessed by postprocedural and follow-up FD-OCT. The clinical endpoint was target lesion revascularization (TLR). TLR was defined as repeated PCI or coronary artery bypass grafting to the lesion in the previously stented segment or in the adjacent 5 mm surrounding it on either side of the stent.

### 2.5. Statistics Analysis

Statistical analysis was performed using JMP 10.0 for Windows (SAS Institute Japan Ltd., Tokyo, Japan). Normally distributed variables were presented as means with standard deviation. The means were compared using the unpaired t-test. Categorical data were presented as percentages. The proportions were compared by the chi-squared test or Fischer's exact test. Differences were considered statistically significant if p <0.05.

## 3. Results

### 3.1. Baseline Clinical and Procedural Characteristics

Patient and procedural characteristics are shown in Tables 1 and 2. The two groups were similar with regard to age, gender, left ventricular function, chronic kidney disease, and hemodialysis. There were fewer patients presenting with ACS who were treated in the ELCA group than the non-ELCA group (0% vs. 25.0%, p<0.01) (Table 1). The number of implanted DES was similar between the groups (55.0% vs. 79.2%, p = 0.09) (Table 2). The maximum pressure of the scoring balloon was similar in the ELCA group and the non-ELCA group (11.0 ± 2.5 atm vs. 12.7 ± 4.0 atm, p = 0.11). Although the mean diameter of scoring balloon and DCB were larger in the ELCA group (3.36 ± 0.35 mm vs. 2.92 ± 0.32 mm, p<0.01, 3.22 ± 0.38 mm vs. 2.87 ± 0.29 mm, p<0.01, respectively), the ratio of scoring balloon and DCB to artery calculated as the maximum balloon diameter divided by RD were similar between the ELCA and the non-ELCA groups (1.36 ± 0.25 vs. 1.23 ± 0.33, p = 0.14, 1.26 ± 0.29 vs. 1.22 ± 0.35, p = 0.66, respectively). Procedural and angiographic successes were achieved in all patients without any complications and bail-out stent implantation after PCI was not required in any of the cases. In the ELCA group, TLR occurred in one patient before 6 months and was excluded from follow-up angiographic and OCT analysis.

### 3.2. Angiographic Results

**Figure 2:**
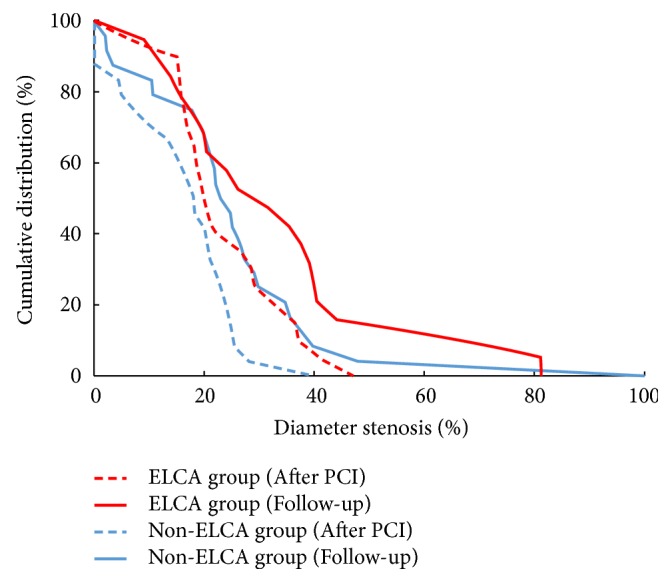
*Cumulative distribution curves of diameter stenosis immediately after percutaneous coronary intervention procedure and at follow-up angiography.* Percent diameter stenosis after PCI was significantly higher in the ELCA group than in the non-ELCA group (p = 0.02), but there was no significant difference between the groups at follow-up (p = 0.21). ELCA, excimer laser coronary atherectomy; PCI, percutaneous coronary intervention.

QCA results are listed in Table 2. In terms of the ISR type, diffuse restenosis in the ELCA group was more frequent than that in the non-ELCA group (55.0% vs. 12.5%, p<0.01). In the ELCA group, MLD increased from 0.61 ± 0.24 to 2.08 ± 0.42 mm after PCI, but had declined to 1.77 ± 0.68 mm at follow-up. In the non-ELCA group, MLD increased from 0.44 ± 0.32 to 2.16 ± 0.42 mm, but declined to 1.89 ± 0.58 mm at follow-up. There was significant greater %DS after PCI in the ELCA group (24.2 ± 10.4% vs. 16.6 ± 10.0%, p = 0.02), but no significant difference between the groups at follow-up (34.5 ± 21.5% vs. 26.4 ± 19.6%, p = 0.21) (Figure 2). Acute gain was 1.50 ± 0.48 mm in the ELCA group and 1.71 ± 0.49 mm in the non-ELCA group, with no significant difference (p = 0.16). In terms of LLL and LLE, ELCA and non-ELCA groups showed comparable results (0.34 ± 0.77 mm vs. 0.26 ± 0.65 mm, p = 0.74, 31.6% vs. 37.5%, p = 0.69, respectively). Regarding binary angiographic restenosis, the ELCA group showed similar result as compared with the non-ELCA group (15.8% vs. 4.2%, p = 0.19).

### 3.3. OCT Results

**Figure 3:**
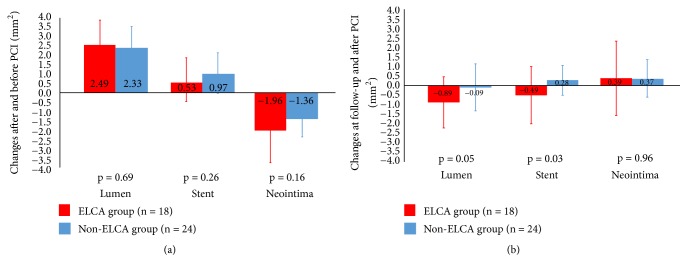
*Comparison of MLCSA, MSCSA, and neointimal CSA between the ELCA group and the non-ELCA group.* The changes in the MLCSA and MSCSA before, after PCI, and at 6 months of follow-up between the ELCA and the non-ELCA group are shown (a, b). (a) The changes after and before PCI in the MLCSA, MSCSA, and neointimal CSA were similar in both groups. (b) A decreased tendency in the MLCSA (-0.89 ± 1.36 mm^2^ vs. -0.09 ± 1.25 mm^2^, p = 0.05) and a significant decrease in the MSCSA was observed between 6-month follow-up and after PCI in the ELCA group (-0.49 ± 1.48 mm^2^ vs. 0.28 ± 0.78 mm^2^, p = 0.03). The changes in the neointimal CSA showed no significant difference (0.39 ± 1.94 mm^2^ vs. 0.37 ± 0.98 mm^2^, p = 0.96). MLCSA, minimum lumen cross-sectional area; MSCSA, minimum stent cross-sectional area; ELCA, excimer laser coronary atherectomy; PCI, percutaneous coronary intervention.

**Figure 4:**
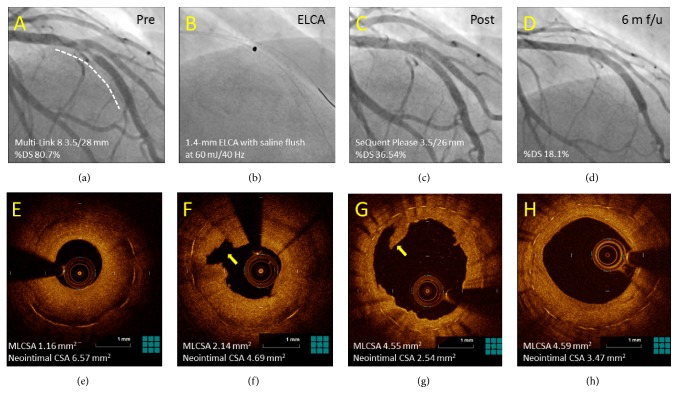
*Representative angiographic and FD-OCT images: dissection and healing.* The angiographic and FD-OCT images are shown before, after the PCI procedure, and at 6 months of follow-up in the ELCA group (a–h). (a) Baseline angiogram showing BMS ISR in the LAD (white dotted line). (b) 1.4-mm ELCA with saline flush at 60 mJ/40 Hz. (c) Final angiogram after DCB dilatation (SeQuent Please 3.5/26 mm inflated at 8 atm for 45 seconds). (d) Six-month follow-up angiogram. (e) Pre-PCI FD-OCT image. (f) Cavities after ELCA (yellow arrow). (g) Flap dissections after PCI (yellow arrow). (h) Completely healed with restoration of smooth luminal contour at 6 months of follow-up. FD-OCT, frequency-domain optical coherence tomography; PCI, percutaneous coronary intervention; ELCA, excimer laser coronary atherectomy; BMS, bare-metal stent; ISR, in-stent restenosis; LAD, left anterior descending artery; DCB, drug-coated balloon; PCI, percutaneous coronary intervention.

FD-OCT results are listed in Table 3 and Figures 3(a)-3(b). The MLCSA, MSCSA, and neointimal CSA before and after PCI were similar in both groups. In terms of percentage of neointimal CSA after PCI, the ELCA group was smaller than that in the non-ELCA group (43.7 ± 13.2% vs. 51.2 ± 10.7%, p = 0.04). However, at 6 months of follow-up, there was similar result in the percentage of neointimal CSA (52.5 ± 19.4% vs. 54.1 ± 15.3%, p = 0.75). The changes after and before PCI in the MLCSA, MSCSA, and neointimal CSA were similar in both groups (Figure 3(a)). In the ELCA group, a decreased tendency in the MLCSA (-0.89 ± 1.36 mm^2^ vs. -0.09 ± 1.25 mm^2^, p = 0.05) and a significant decrease in the MSCSA was observed between 6-month follow-up and after PCI as compared with those in the non-ELCA group (-0.49 ± 1.48 mm^2^ vs. 0.28 ± 0.78 mm^2^, p = 0.03) (Figure 3(b)). The changes in the neointimal CSA at follow-up and after PCI showed no significant difference in the ELCA and the non-ELCA groups (0.39 ± 1.94 mm^2^ vs. 0.37 ± 0.98 mm^2^, p = 0.96). Although the number of dissections after PCI tended to be less in the ELCA group as compared with the non-ELCA group (5.8 ± 3.6 vs. 9.0 ± 6.9, p = 0.07), there was no significant difference between the groups in the number of dissections at 6 months of follow-up (0.1 ± 0.3 vs. 0.2 ± 0.4, p = 0.24). Representative angiographic and FD-OCT images in the ELCA group are shown before, after the PCI procedure, and at 6 months of follow-up in Figure 4. Several dissections were observed immediately after PCI. Complete healing of the dissection with restoration of smooth luminal contour and without irregularities was observed in both groups at 6 months of follow-up.

### 3.4. Clinical Outcome

In-hospital major adverse cardiac events, defined as a composite outcome of cardiac death, and nonfatal MI, did not occur between the groups. The ELCA group and non-ELCA group showed comparable rates of TLR (11.1% vs. 11.4%, p = 0.85) at 1 year. There was no difference in time to TLR between the ELCA group and the non-ELCA group (184 ± 59 days vs. 293 ± 81 days; p = 0.52).

## 4. Discussion

The major findings of this study are (1) diffuse restenosis and %DS after PCI were greater in the ELCA group; (2) LLL and binary restenosis had comparable results in the ELCA and the non-ELCA group; (3) the changes in the MSCSA at follow-up and after PCI showed significant decrease in the ELCA group; (4) the changes in the neointimal CSA at follow-up and after PCI showed no significant difference between the two groups; and (5) TLR showed comparable rates in both groups. These results may suggest the possible benefit of ELCA for diffuse restenosis.

### 4.1. Efficacy of DCB

Our results showed that there was no significant difference between the ELCA and non-ELCA groups in LLL, and the changes in the neointimal CSA at follow-up and after PCI showed no significant difference in both groups. Moreover, the number of dissections at 6 months of follow-up was not significantly different between the groups. Although the underlying pathophysiological mechanism of treatment with DCB remains unclear, adequate delivery of paclitaxel is important for preventing superficial effects on endothelial cells to enable dissections to heal faster and suppress neointimal proliferation as well as migratory processes simultaneously [11–13]. A previous study reported that DCB treatment highly reduced LLL than plain old balloon angioplasty for ISR lesions (0.11 ± 0.44 mm vs. 0.80 ± 0.79, p = 0.001) [14]. Mehran et al. evaluated the efficacy of ELCA plus POBA as compared with POBA alone with intravascular ultrasound (IVUS) [5]. As compared with IVUS, OCT has demonstrated superiority with improved image resolution and contrast. OCT provides accurate measurement of vessel sizing and detection of thrombus, intimal hyperplasia, intimal tears, and dissection. Fukushima et al. showed that the number of dissections at 6 months of follow-up decreased with DCB treatment as compared without DCB by OCT [15], which is probably related to the repair of dissections, vascular healing, plaque regression, and positive vessel remodeling. Therefore, in this study, similar changes in the neointimal CSA, as well as the number of dissections at 6 months of follow-up, indicated the efficacy of DCB.

### 4.2. ELCA for Diffuse ISR

In our study, despite the more frequent diffuse restenosis and greater %DS after PCI in the ELCA group, there was no significant difference in %DS at 6-month follow-up, LLL, and binary restenosis between the ELCA and non-ELCA group. Diffuse restenosis has been shown to be associated with a higher rate of recurrent rerestenosis and poor prognosis than focal restenosis in the bare-metal stent (BMS) and DES [16, 17]. The importance of residual %DS <20% as a fundamental requirement of optimized DCB procedure has been demonstrated [18]. The ISAR-DESIRE 4 (Intracoronary Stenting and Angiographic Results: Optimizing Treatment of Drug-Eluting Stent In-Stent Restenosis) trial showed that predilation with a scoring balloon resulted in significantly smaller %DS at 6-month angiographic follow-up than balloon angioplasty alone (35.0 ± 16.8% vs. 40.4 ± 21.4%, p = 0.047) [19]. However, in clinical practice, lesion preparation before DCB procedure is not always easily performed by balloon angioplasty alone, especially for diffuse ISR or previously implanted stent with underexpansion. This was one of the possible reasons that larger balloon may be required to dilate the lesion in the ELCA group, despite similar RD in both groups. In this study, the percentage of neointimal CSA after PCI was smaller in the ELCA group (43.7 ± 13.2% vs. 51.2 ± 10.7%, p = 0.04). Luminal gain after treatment with ELCA for ISR might be achieved by a combination of neointimal tissue extrusion and stent expansion by disrupting the plaque behind the stent strut [20–23]. Lee T et al. showed that ELCA is effective for stent underexpansion disrupting peri-stent calcium as assessed by OCT [24]. Furthermore, the number of dissections after PCI tended to be less in the ELCA group as compared with the non-ELCA group, which seemed to be safe and effective for avoiding bail-out stenting by ELCA.

In contrast to acute results, the ELCA group and non-ELCA group showed comparable rates of TLR at 1 year. Our study showed that the result of the LLL, the neointimal CSA, and the percentage of neointimal area at follow-up did not have significant differences in both groups. Additionally, a decreased change at follow-up and after PCI in the MLCSA and MSCSA was observed in the ELCA group (Figure 3(b)). The deterioration of MSCSA by ELCA might not be clearly explained, but laser-induced shock waves and forceful expansion of vapor bubbles into the lesion beneath the stent struts could have caused not only acute vessel expansion, but also inflammation or proliferation outside the stent. Thus, with regard to lesion modification before DCB treatment, ELCA might be partially offset by the suppression of neointimal proliferation with DCB treatment but was certainly effective for the treatment of diffuse ISR lesions.

### 4.3. Study Limitations

The main limitation of our study is the absence of randomization towards the treatment strategy. A larger sample size, multivariate analysis, and longer follow-up are needed to confirm the conclusions. Selection bias that diffuse restenosis was more frequent in the ELCA group might cause the clinical and angiographic results because the minimum lumen site in re-ISR lesions might not always correspond to those in the primary ISR lesions. The relationship between BMS ISR and DES ISR after treatment with ELCA could not be respectively analyzed because of the small sample size. The impact of different ELCA catheter sizes and further modification with noncompliant balloon before DCB treatment on angiographic or OCT findings was not evaluated. The time after stent implantation, the presence of stent fracture, or inadequate stent apposition was not evaluated in our study. In addition, qualitative analysis and three-dimensional volumetric analysis of the entire lesion length were not performed in this study. Another limitation is that the impact of morphology of dissection on angiographic and OCT results at follow-up was not assessed, as well as the degrees of longitudinal and circumferential distribution.

## 5. Conclusion

By OCT analysis, although ELCA before DCB treatment might partially offset the suppression of neointimal proliferation because of a decreased change in the stent and lumen, ELCA had a possible benefit with regard to lesion modification for diffuse restenosis.

## Figures and Tables

**Table 1:** Baseline patient characteristics.

	ELCA group	Non-ELCA group	p value
	(n = 18)	(n = 24)	

Age, means, y	68.4 ± 9.7	71.5 ± 8.4	0.27
Male gender, %	14 (77.8)	20 (83.3)	0.65
Smoking, %	9 (50.0)	3 (12.5)	<0.01
Hypertension, %	12 (66.7)	21 (87.5)	0.10
Dyslipidemia, %	16 (88.9)	22 (91.7)	0.76
Diabetes mellitus, %	7 (38.9)	13 (54.2)	0.33
Chronic kidney disease, %	4 (22.2)	11 (45.8)	0.11
Hemodialysis, %	0 (0)	0 (0)	NS
Previous MI, %	7 (38.9)	17 (70.8)	0.04
Previous CABG, %	0 (0)	2 (8.3)	0.13
EF, %	59.3 ± 12.8	60.8 ± 12.4	0.71
Clinical presentation			<0.01
ACS, %	0 (0)	6 (25.0)	
Non-ACS, %	18 (100)	18 (75.0)	

Continuous variables expressed as mean ± standard deviation; categorical variables, as number (percentage).

ELCA, excimer laser coronary atherectomy; MI, myocardial infarction; CABG, coronary artery bypass graft; EF, left ventricular ejection fraction; ACS, acute coronary syndromes.

**Table 2:** Angiographic and procedural findings.

	ELCA group	Non-ELCA group	p value
	(n = 18)	(n = 24)	

Number of treated segments, n	20	24	
Target vessel			
LMCA, %	0 (0)	0 (0)	NS
LAD, %	10 (50.0)	17 (70.8)	0.16
LCX, %	2 (10.0)	3 (12.5)	0.79
RCA, %	8 (40.0)	4 (16.7)	0.08
Bypass graft, %	0 (0)	0 (0)	NS
Previous stent type			0.09
BMS, %	9 (45.0)	5 (20.8)	
DES, %	11 (55.0)	19 (79.2)	
Restenosis morphology			
Focal margin, %	1 (5.0)	6 (25.0)	0.06
Focal body, %	4 (20.0)	9 (37.5)	0.20
Multifocal, %	3 (15.0)	4 (16.7)	0.88
Diffuse, %	11 (55.0)	3 (12.5)	<0.01
Proliferative, %	0 (0)	0 (0)	NS
Occlusive, %	1 (5.0)	4 (16.7)	0.21
Scoring balloon			
Balloon diameter, mm	3.36 ± 0.35	2.92 ± 0.32	<0.01
Inflation pressure, atm	11.0 ± 2.5	12.7 ± 4.0	0.11
Balloon-to-artery ratio	1.36 ± 0.25	1.23 ± 0.33	0.14
DCB			
Mean diameter, mm	3.22 ± 0.38	2.87 ± 0.29	<0.01
Total length, mm	23.7 ± 5.1	28.0 ± 14.4	0.20
Inflation pressure, atm	9.2 ± 3.0	8.6 ± 2.6	0.50
Inflation time, sec	49.8 ± 7.7	54.2 ± 10.0	0.11
Balloon-to-artery ratio	1.26 ± 0.29	1.22 ± 0.35	0.66
*QCA analysis*			
Preprocedure			
Minimal luminal diameter, mm	0.61 ± 0.24	0.44 ± 0.32	0.06
Diameter stenosis, %	68.4 ± 25.1	82.2 ± 12.6	0.02
Reference vessel diameter, mm	2.54 ± 0.57	2.45 ± 0.48	0.58
Lesion length, mm	17.4 ± 11.1	23.0 ± 10.7	0.11
Postprocedure			
Minimal luminal diameter, mm	2.08 ± 0.42	2.16 ± 0.42	0.58
Diameter stenosis, %	24.2 ± 10.4	16.6 ± 10.0	0.02
Acute gain, mm	1.50 ± 0.48	1.71 ± 0.49	0.16
Follow-up			
Minimal luminal diameter, mm	1.77 ± 0.68	1.89 ± 0.58	0.53
Diameter stenosis, %	34.5 ± 21.5	26.4 ± 19.6	0.21
Late lumen loss, mm	0.34 ± 0.77	0.26 ± 0.65	0.74
Late lumen enlargement, %	6 (31.6)	9 (37.5)	0.69
Binary restenosis, %	3 (15.8)	1 (4.2)	0.19

Continuous variables expressed as mean ± standard deviation; categorical variables, as number (percentage). Balloon-to-artery ratio was calculated as the ratio of the maximum balloon diameter by reference vessel diameter.

ELCA, excimer laser coronary atherectomy; LMCA, left main coronary artery; LAD, left anterior descending artery; LCX, left circumflex artery; RCA, right coronary artery; BMS, bare-metal stent; DES, drug-eluting stent; DCB, drug-coated balloon; QCA, quantitative coronary angiography.

**Table 3:** Optical coherence tomography analysis.

	ELCA group	Non-ELCA group	p value
	(n = 18)	(n = 24)	

*Pre-PCI procedure*			
MLCSA, mm^2^	1.37 ± 0.93	1.02 ± 0.31	0.13
MSCSA, mm^2^	6.46 ± 2.14	5.80 ± 1.92	0.32
Neointimal CSA, mm^2^	5.09 ± 2.16	4.75 ± 1.85	0.60
Percentage of neointimal area, %	77.9 ± 13.2	80.6 ± 6.6	0.44
*After ELCA procedure*			
MLCSA, mm^2^	1.63 ± 0.91	N/A	
MSCSA, mm^2^	6.67 ± 2.19	N/A	
Neointimal CSA, mm^2^	5.03 ± 2.22	N/A	
Percentage of neointimal area, %	74.1 ± 13.5	N/A	
*After DCB procedure*			
MLCSA, mm^2^	3.85 ± 1.11	3.42 ± 1.14	0.21
MSCSA, mm^2^	6.99 ± 2.14	7.17 ± 2.26	0.78
Neointimal CSA, mm^2^	3.13 ± 1.44	3.75 ± 1.60	0.19
Percentage of neointimal area, %	43.7 ± 13.2	51.2 ± 10.7	0.04
*Follow-up*			
MLCSA, mm^2^	3.07 ± 1.37	3.33 ± 1.18	0.51
MSCSA, mm^2^	6.66 ± 1.92	7.44 ± 1.98	0.20
Neointimal CSA, mm^2^	3.59 ± 1.91	4.12 ± 1.84	0.36
Percentage of neointimal area, %	52.5 ± 19.4	54.1 ± 15.3	0.75
*Changes ELCA and pre PCI*			
ΔMLCSA, mm^2^	0.67 ± 0.42	N/A	
ΔMSCSA, mm^2^	0.21 ± 0.99	N/A	
ΔNeointimal CSA, mm^2^	-0.06 ± 1.13	N/A	
*Changes after PCI and ELCA*			
ΔMLCSA, mm^2^	2.22 ± 1.19	N/A	
ΔMSCSA, mm^2^	0.32 ± 1.07	N/A	
ΔNeointimal CSA, mm^2^	-1.90 ± 1.48	N/A	
*Changes after and pre PCI*			
ΔMLCSA, mm^2^	2.49 ± 1.31	2.33 ± 1.13	0.69
ΔMSCSA, mm^2^	0.53 ± 1.38	0.97 ± 1.02	0.26
ΔNeointimal CSA, mm^2^	-1.96 ± 1.56	-1.36 ± 0.92	0.16
*Changes at follow-up and after PCI*			
ΔMLCSA, mm^2^	-0.89 ± 1.36	-0.09 ± 1.25	0.05
ΔMSCSA, mm^2^	-0.49 ± 1.48	0.28 ± 0.78	0.03
ΔNeointimal CSA, mm^2^	0.39 ± 1.94	0.37 ± 0.98	0.96
*Number of dissections*			
After PCI	5.8 ± 3.6	9.0 ± 6.9	0.07
Follow-up	0.1 ± 0.3	0.2 ± 0.4	0.24
Changes at follow-up and after PCI	-5.7 ± 3.5	-8.8 ± 6.8	0.09

Continuous variables expressed as mean ± standard deviation; categorical variables, as number (percentage).

ELCA, excimer laser coronary atherectomy; PCI, percutaneous coronary intervention; MLCSA, minimum lumen cross-sectional area; MSCSA, minimum stent cross-sectional area; CSA, cross-sectional area; DCB, drug-coated balloon.

## Data Availability

The data used to support the findings of this study are available from the corresponding author upon request.
